# The effect of lifestyle intervention and depression symptoms on binge eating and relation of binge eating to gestational weight gain and child birth weight in the UPBEAT cohort of pregnant women living with obesity

**DOI:** 10.1371/journal.pone.0332569

**Published:** 2025-09-18

**Authors:** Sam Burton, Francesca Solmi, Kathryn V. Dalrymple, Angela C. Flynn, Keith M. Godfrey, Lucilla Poston, Abigail Easter

**Affiliations:** 1 School of Psychology, Faculty of Health, Liverpool John Moores University, Liverpool, United Kingdom; 2 Department of Women’s and Children’s Health, School of Life Course and Population Sciences, Faculty of Life Sciences & Medicine, King’s College London, London, United Kingdom; 3 Division of Psychiatry, Faculty of Brain Sciences, University College London, London, United Kingdom; 4 Department of Population Health Sciences, School of Life Course and Population Sciences, Faculty of Life Sciences and Medicine, King’s College London, London, United Kingdom; 5 Department of Nutritional Sciences, School of Life Course and Population Sciences, Faculty of Life Sciences and Medicine, King’s College London, London, United Kingdom; 6 MRC Lifecourse Epidemiology Centre and NIHR Southampton Biomedical Research Centre, University of Southampton and University Hospital Southampton NHS Foundation Trust, Southampton, United Kingdom; Trinium Woman’s Hospital, KOREA, REPUBLIC OF

## Abstract

Binge eating is one of the most prevalent eating disorder behaviours in pregnancy, its risk factors and association with pregnancy-related outcomes has sparsely researched in this population. This study aimed to investigate: (hypothesis 1) the effectiveness of a lifestyle intervention in reducing binge eating; (hypothesis 2) the association between depressive symptoms and binge eating behaviours throughout the perinatal period; and (hypothesis 3) the association between binge eating, gestational weight gain and birthweight in a cohort of pregnant women with obesity. This is a planned secondary analysis of the UK Pregnancies Better Eating and Activity Trial (UPBEAT) randomized controlled trial. Exposures were trial arms (hypothesis 1); depressive symptoms (hypothesis 2); and number of weekly binge eating episodes and binge eating behaviours (hypothesis 3). Outcomes were number of weekly binge eating episodes and binge eating behaviours and cognitions (hypotheses 1 and 2), gestational weight gain and child’s birthweight (hypothesis 3). There was no evidence that the UPBEAT intervention was effective in reducing number of weekly binge eating behaviours (IRR .942; 95%CI .756, 1.174) or binge eating behaviours (IRR 1.005; 95%CI .861, 1.174). Increased levels of depressive symptoms were associated with a higher number of binge eating behaviours (IRR 1.031; 95%CI 1.015, 1.048) and its associated features (IRR 1.030; 95%CI 1.019, 1.041). There was evidence that more frequent binge eating behaviours lead to greater increase in gestational weight gain. (coefficient = .614; 95%CI .264, .964). There is a need for holistic interventions that promote maternal mental health and address binge eating behaviours. More work is required in the field to understand which interventions would prove efficacious.

## 1. Introduction

Pregnancy involves profound psychological, social, and biological changes that influence eating behaviour and body perceptions [1,2]. An estimated 1.5–7.6% of women are affected by an eating disorder (ED) during pregnancy [3–6]. Binge Eating Disorder (BED) and Binge Eating Behaviours have been identified as some of the most prevalent types of ED and behaviours during pregnancy, with evidence suggesting remission of such behaviours may be heightened during the perinatal period but not for all (Bulik et al., 2007), this remission is not universal and may not be sustained without appropriate support. The current evidence base is dispersed in respect to methodologies, with a need for empirical research examining both risk factors and outcomes for BED during pregnancy to allow the development of suitable interventions to maintain remission [7].

Little is known about how to treat BED in pregnancy, nor in the general population. Previous lifestyle interventions have shown mixed success in changing eating behaviours and nutritional intake in non-pregnant participants [8–11] and no previous work has examined the influence of lifestyle interventions on disordered eating or ED’s within a pregnant population. Recent reviews have highlighted while treatments for BED have been effective at addressing certain components, overall long term efficacy and implementation is minimal [12,13].

Although the aetiology of binge eating is unclear [14], environmental triggers and stressful experiences have been proposed to play a vital role in its onset and maintenance [15,16], along with an inability to discern between adequate versus excessive weight gain [17]. Thoughts of being overweight, depression and over-valuation of weight have been associated with higher incidence of BED over time [1]. Previous history of an ED prior to pregnancy has been associated with increased recurrence of ED and depression during a subsequent pregnancy [18,19], with depression predicting greater engagement in binge eating behaviours [1,20]. Given how up to 21.7% of women are estimated to suffer with depression during pregnancy [21,22], no research has examined it’s association with BED throughout pregnancy.

A growing body of evidence supports the presence of an association between binge eating behaviours, gestational weight gain (GWG) and birth outcomes [23–25]. However, there is limited work [18] examining such factors while accounting for maternal depression. Maternal depression is associated with reduced birthweight, stillbirth, small gestational age and perinatal complications [26]. The relationship between binge eating and GWG have been inconsistent, potentially due to not accounting for maternal depression (Hecht et al., 2020). However, few studies have addressed these interrelated factors longitudinally during pregnancy and their effect on gestational outcomes.

Pregnant women who display behaviours associated with BED are at a greater risk of excessive GWG compared to those without ED [27–29] yet this association has not been consistently supported due to the rarity of it’s investigation [30]. Recent reports suggest an average increase in GWG of 3.74 kg for women that experience loss of control (over eating behaviour) during pregnancy compared to those who do not [23], which is similar to estimates observed in women with overweight or obesity [24] and gestational diabetes [31]. Excessive GWG is associated with obstetric complications [32] and adverse maternal and perinatal outcomes such as pre-term births and large for gestational age [33,34]. Therefore, if binge eating is a risk factor for the latter, interventions targeting binge eating could also improve obstetric outcomes.

The aim of the current study was to investigate the associations between binge eating behaviours, depression, GWG and birth outcomes in a secondary analysis of data from the (UK Pregnancies Better Eating and Activity Trial (UPBEAT) randomised controlled trial. UPBEAT was a multicentre randomised controlled trial of a complex behavioural intervention including health trainer support to increase physical activity and improve diet in women living with obesity [35]. Women received support to achieving SMART goals (e.g., specific, measurable, achievable, relevant, time specific) at each session, along with advice on self-monitoring, identification and problem-solving barriers to behaviour change. While women in the antenatal group attended routine antenatal appointments throughout their pregnancy. This is particularly salient given the lack of research on these conditions and their treatment during the perinatal period. Specifically, we hypothesised that:

1. Participation in the UPBEAT intervention would be associated with a decrease in binge eating behaviours in the perinatal period.
2. Greater depressive symptoms would be longitudinally associated with greater binge eating behaviours and cognitions both through the antenatal and postnatal period.
3. Greater binge eating behaviours and cognitions during pregnancy would be associated with greater maternal GWG and child’s birth weight.

## 2. Materials and methods

This was a pre-planned secondary analysis of the UPBEAT study data. The study was conducted according to the guidelines of the Declaration of Helsinki, and approved by the NHS Research Ethics Committee (UK Integrated Research Application System, reference 09/H0802/5).

### Sample

**Fig 1:**
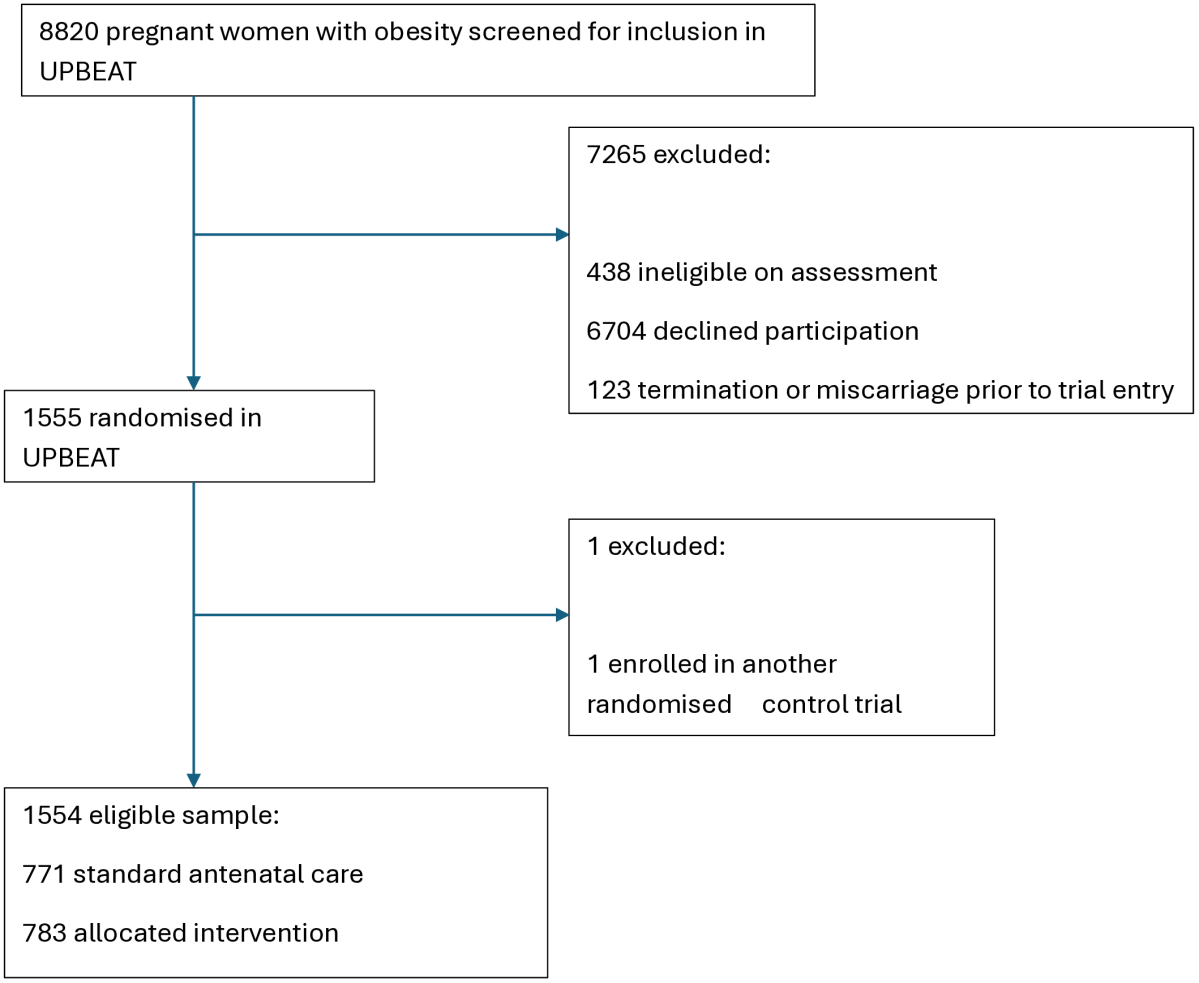
Participant flow.

The UPBEAT study recruited 1555 women from eight UK National Health Service (NHS) hospital trusts between March 2009 and June 2014. Data was accessed on the 1^st^ of March 2022 and was fully anonymised, data can be requested from UPBEAT trial. Women were recruited if they were between 15–18 weeks’ gestation, had a body mass index (BMI) ≥30 kg/m^2^, were over the age of 16 years old, and had a singleton pregnancy. Exclusion criteria were lack of informed consent, use of metformin, and suffering from at least one pre-pregnancy medical condition that may influence appetite and eating outside of a typical pregnancy [35] (see S1 Table). One woman was excluded from our analysis due to being enrolled in another RCT [36], leaving a sample of 1554 (see Fig 1). In this sample we included participants who had data available on binge eating behaviours for at least 1 of the time points other than the baseline assessment at 15–18 weeks’ gestation.

Women who were randomised to the intervention received a maximum of eight weekly one-hour sessions aimed at supporting physical activity and dietary goals [35]. Women in the control group received standard antenatal care (NICE, 2021). Full details of the UPEBAT intervention and standard antenatal care are reported in prior UPBEAT publications (Poston et al., 2015). All women gave informed consent prior to their inclusion in the study. The trial arm was used as an exposure in the analysis for hypothesis 1.

### Measures

The questionnaires were administered at study baseline (15^+0 days^-18^+6 days^ weeks’ gestation), and again at 27^+0 days^-28 ^+6 days^ weeks’ gestation (end of the UPBEAT intervention), 34^+0 days^-36^+0 days^ weeks’ gestation, and 6-month postpartum follow-up visits, using self-report forms.

#### Binge eating behaviour and cognitions.

Binge eating behaviours (e.g., overeating) and cognitions (e.g., loss of control over eating behaviours) were measured at all time-points using the validated Eating Disorder Diagnostics Scale (EDDS) [37]. Presence of compensatory behaviours, necessary to derive a diagnosis of BED, was only measured at the baseline assessment. At the baseline assessment, (15^+0 days^-18^+6 days^ weeks), EDDS questions assessed lifetime occurrence of binge eating-related behaviours. Whereas at follow-up assessments questions referred to the time period “since the last research visit” and asked to provide an average amount of overeating episodes coupled with loss of control within a week. Below we detail how we defined BED diagnosis in the sample as well as presence of binge eating behaviours and their associated features.

**Binge eating disorder**: We considered participants as having a probable diagnosis of Diagnostic and Statistical Manual of Mental Disorders 4 Binge Eating Disorder (DSM-IV BED) at baseline if they:

a) experienced regular eating binges marked by a perceived loss of control and the consumption of a large amount of food as indexed by a response of yes to; AND
b) endorsed at least three of the features that may be associated with binge eating (i.e., Eat much more rapidly than normal, eating when not hungry, eating in secret, eating until uncomfortably full, feeling guilty after overeating); AND
c) experienced marked distress regarding binge eating AND
d) did not engage in any purging or non-purging compensatory behaviours, e.g., self-induced vomiting, laxative use, or excessive exercise

### Binge eating behaviours and cognitions

Binge eating behaviours and cognitions were assessed on the following two composite measures:

**Number of binge eating episodes:** Number of binge eating behaviour instances present [38,39]. Binge eating was defined as experiencing a loss of control leading to the consumption of a large amount of food. In line with EDDS coding, we derived a variable specifying the number of binge eating episodes experienced in a week; values ranged from 0 (i.e., no binge eating) to 7 (at least 7 episodes of binge eating). Comparable definitions of binge eating have been widely used in population samples [40,41]; [42–45].

**Number of associated features of binge eating endorsed:** We additionally created a count variable ranging from 0 to 5 indicating the number of binge eating behaviours (e.g., eat much more rapidly than normal, eating in secret, eating a large amount of food when you don’t feel hungry) experienced by the participant on a *weekly* basis. When coded as a binary variable the cut off for endorsing binge eating was classified as displaying three or more binge endorsement behaviours, as defined in the EDDS.

We used the ‘binge eating’ and ‘associated features of binge eating’ as the outcome variable for hypotheses 1 and 2, and the exposure in hypothesis 3 and exploratory analysis.

#### Depressive symptoms.

At all time points, symptoms of depression were measured using the validated Edinburgh Postnatal Depression Scale (EPDS) which covers symptoms experienced in the two weeks preceding assessment [46]. Scores were calculated as linear (out of a maximum of 30) and binary outcomes (≥13 or <13 as a validated cut-off score for lower or higher levels of depressive symptoms respectively) [47,48]. Depressive symptoms were the exposure for hypothesis 2.

#### Gestational weight.

Gestational weight was measured at baseline and subsequent antenatal follow ups. Mothers were measured and weighed by a healthcare professional, with the difference in baseline weight calculated at each follow up assessment. This was the outcome variable in hypothesis 3.

#### Birth weight.

Upon delivery the baby was measured and weighed and customised birthweight centiles with Gestation Related Optimal Weight (GROW) software, version 6.7.5.1 (Gestation Network, Perinatal Institute, Birmingham, UK) were calculated. This was the outcome variable in hypothesis 3

#### Confounders.

Lifetime binge eating instances and lifetime features associated with binge eating were adjusted for in the analysis pertaining to hypothesis 1. Analyses pertaining to hypotheses 2 and 3 were adjusted for potential confounders of the associations under investigation, with depressive symptoms additionally adjusted for in analysis for hypothesis 3. These were:

1. Baseline maternal weight: the participants’ weight in kg, measured at baseline 15^+0 days^-18^+6 days^ weeks gestation.
2. Deprivation: measured using the index of multiple deprivation (IMD) associated with participants residential postcode and expressed as fifths based on the IMD distribution in the English population [49]. Adjusted values were used for Scottish addresses [50].
3. Ethnicity: participants reported their main ethnicity, which was categorised as Asian, Black, White, or Other in line with Office for National Statistics categorisations.

Analyses pertaining to hypothesis 1 and 2, were also adjusted for:

4. Lifetime features associated with binge eating: this was calculated from the EDDS sub-scale on binge eating endorsement, when worded in respect to the participants lifetime history of eating behaviours.
5. Lifetime binge eating instances: this was calculated from the EDDS sub-scale on binge eating loss of control, when worded in respect to the participants lifetime history of eating behaviours.
6. Depressive symptoms: calculated from the EPDS as a linear outcome.

#### Covariates.

1. Randomisation to the intervention: intervention versus control group (hypothesis 2).

### Statistical analysis

Of the binge eating data a proportion of 39% was missing across all timepoints. To handle missing data for outcome variables multi-level models were used applying maximum-likelihood methods given that outcome data are assumed to be missing at random yielding robust results [51–53]. Descriptives of sample demographics and the prevalence of binge eating behaviours across time points, were analysed presenting means (standard deviations), medians (inter-quartile range) and counts (percentages).

Hypothesis 1: To investigate the effect of the intervention on number of associated binge eating behaviours and cognitions, and number of binge eating episodes, multilevel Poisson regression models, with random intercept for participant and a time variable indicating follow up assessment as a fixed effect. Adjustments were made for lifetime number of binge eating episodes measured at baseline and lifetime number of associated features of binge eating endorsed measured at baseline.

Hypothesis 2: To investigate the effect of depressive symptoms on number of associated binge eating behaviours and congitions, and number of binge eating episodes, multilevel Poisson regression models, with random intercept for participant and a time variable indicating follow up assessment as a fixed effect. Univariable and multivariable models adjusting for baseline weight, deprivation, ethnicity, treatment arm, lifetime features associated with binge eating measured at baseline and lifetime binge eating instances measured at baseline.

Hypothesis 3: To investigate the association between binge eating exposures, and GWG and birthweight we used a multilevel linear regression model nesting repeated time point within participants. In our models we included a random intercept on participants and a time variable indicating follow up assessment as a fixed effect. A two-level model was compared to a single level model (with no random intercept of participant) using a Chi-squared test to determine goodness of fit, if the models were significantly different the two-level model was used going forward.

To investigate the outcome of GWG we used multilevel regression models, modelling outcomes at repeated time points for individual participants, we fitted a random intercept of time point in the models.

R was used for data manipulation and analysis using the following packages, lm, lmer, lavaan, tidyverse, naniar, jtools and afex.

## 3. Results

### Sample characteristics

The characteristics of the sample of 1554 women are presented in Table 1. Mean sample age was 30.5 years (standard deviation (SD) 5.5) and median BMI was 35 (Inter Quartile Range (IQR) 32.8–38.5). Over 75% of participants lived in the two most deprived fifths of IMD and 63% of participants were of white ethnicity. There was no significant difference between trial arms on variables presented in Table 1.

**Table 1:** Characteristics of the sample. Significance testing between arms were conducted as followed Chi-Squared for count data, T-test for means, and Kruskal Wallis for medians.

	Total (n = 1554)	Standard (n = 771)	Intervention (n = 783)	*P value*
**Age** **Mean(Standard Deviation (SD))**	30.5 (5.5)	30.5 (5.6)	30.5 (5.5)	.651
**BMI** **Median (IQR)**	35.0 (32.8-38.5)	35.2 (33.0-38.4)	35.0 (32.6-38.7)	.880
	**N (%)**	**n(%)**	**n(%)**	
**Ethnicity**				.943
** White**	973 (62.6%)	483 (62.7%)	490 (65.6%)	
** Black**	401 (25.8%)	199 (25.8%)	202 (25.8%)	
** Asian**	95 (6.1%)	48 (6.2%)	47 (6.1%)	
** Other**	85 (5.5%)	41 (5.3%)	44 (5.6%)	
**Parity**				.442
** Primiparous**	674 (43.4%)	338 (43.8%)	336 (43.0%)	
** Multiparous**	880 (56.3%)	433 (56.2%)	447 (57.0%)	
**Smoker status**				
** Current**	108 (7.0%)	60 (7.8%)	48 (6.1%)	.229
**IMD**				.073
** 1 (Least)**	65 (4.2%)	36 (4.7%)	29 (3.7%)	
** 2**	103 (6.6%)	44 (5.7%)	59 (7.6%)	
** 3**	177 (11.4%)	84 (10.9%)	93 (12.0%)	
** 4**	533 (34.3%)	288 (37.4%)	245 (31.5%)	
** 5 (Most)**	670 (43.1%)	319 (41.5%)	352 (45.2%)	
**Baseline EPDS** **(/30 )**				
** Median (IQR)**	6 (3–10)	6 (3–10)	6 (3–10)	.488
** Missing n (%)**	195 (12.6%)	102 (13.2%)	93 (11.9%)	
** ≥13**	260 (16.7%)	81 (10.5%)	179 (22.9%)	
** <13**	1192 (76.7%)	588 (76.3%)	604 (77.1%)	
**Baseline** **Binge Endorsement**				
** Median (IQR)**	3 (2–5)	3 (2–5)	3 (2–5)	.813
** Missing n (%)**	208 (13.4%)	394 (22.2%)	114 (14.6%)	
**Baseline Binge Loss of Control**				
** Median (IQR)**	1 (1–2)	1 (1–2)	1 (1–2)	.966
** Missing n (%)**	192 (12.4%)	96 (12.5%)	96 (12.3%)	

### Prevalence of binge eating disorder at baseline

A total of 23 (1.48%) participants met full criteria for BED. Given the low prevalence of BED in the sample, we decided to use only number of binge eating episodes and associated features of binge eating in the subsequent analysis in order to retain sufficient statistical power.

Table 2 shows the prevalence of features associated with binge eating and number of binge eating instances for the sample, at baseline, 28 weeks of pregnancy, 36 weeks of pregnancy and 6-weeks post-partum. S1 Table shows data from Table 2 broken down by trial arm, chi-squared tests indicated no significant difference between arms. Demographics variables did not significantly differ between the trial arms within Table 1 [36], and for binge-eating behaviours and EPDS there were no significant differences within S1 Table.

**Table 2:** Prevalence of binge endorsement, binge loss of control, n (%) for each timepoint. A is lifetime binge eating behaviours, b binge eating behaviours in the previous month.

		Time Point			
		Baseline^a^	28 Weeks^b^	32 Weeks^b^	6 Weeks Post-partum^b^
Binge eating variable		n(%)	n(%)	n(%)	n(%)
**Number of binge eating episodes**	0	1024 (75.62%)	1073 (93.63%)	919 (94.94%)	564 (79.77%)
	1	137 (10.12%)	39 (3.40%)	35 (3.62%)	65 (9.19%)
	2	109 (8.05%)	25 (2.18%)	8 (.83%)	44 (6.22%)
	3	58 (4.28%)	6 (.52%)	4 (.41%)	25 (3.54%)
	4	26 (1.93%)	3 (.26%)	2 (.21%)	9 (1.27%)
	**Yes**	**330 (24.37%)**	**73 (6.37%)**	**49 (5.06%)**	**143 (20.23%)**
	**No**	**1024 (75.33%)**	**1073 (93.63%)**	**919 (94.94%)**	**564 (79.77%)**
	N	1354	1146	968	707
	Missing	200 (12.87%)	154 (11.85%)	124 (11.36%)	13 (1.81%)
	Drop out		254 (16.34%)	462 (29.73%)	834 (53.67%)
**Number of associated features of binge eating endorsed**	0	962 (70.84%)	1064 (92.68%)	912 (94.21%)	552 (77.97%)
	1	51 (3.76%)	10 (.87%)	11 (1.14%)	22 (3.12%)
	2	64 (4.71%)	21 (1.83%)	11 (1.14%)	30 (4.24%)
	3	85 (6.26%)	25 (2.18%)	9 (.93%)	31 (4.38%)
	4	86 (6.33%)	12 (1.05%)	15 (1.55%)	32 (4.52%)
	5	110 (8.10%)	16 (1.39%)	10 (1.03%)	41 (5.79%)
**Endorsement of three or more binge eating behaviours**	**Yes**	**281 (20.75%)**	**53 (4.62%)**	**34 (3.51%)**	**104 (14.69%)**
	**No**	**1077 (79.25%)**	**1095 (95.38%)**	**934 (96.49%)**	**604 (85.31%)**
	N	1358	1148	968	708
	Missing	196 (12.61%)	152 (11.69%)	124 (11.36%)	12 (1.67%)
	Drop out		254 (16.34%)	462 (29.73%)	834 (53.67%)

### Hypothesis 1: The effect of UPBEAT intervention on binge eating behaviours and cognitions

Contrary to hypothesis 1, there was no evidence that participating in the UPBEAT intervention was associated with a reduction in number of binge eating episodes (IRR: 0.94, 95% 0.75 to 1.17) and of associated features of binge eating (IRR 1.01, 95% CI 0.86 to 1.17) (Table 3).

**Table 3:** Multilevel Poisson regression models, of associations between intervention arm and binge endorsement, and binge loss of control. Lifetime features associated with binge eating and binge eating episodes was fitted for each respective model as an outcome Reporting Incidence Rate Ratio (IRR) and 95% Confidence Intervals (CI). The table also shows the time point and intervention interaction on both binge endorsement and loss of control, reporting the respective p values.

	Intervention Arm		Time point & Intervention Arm Interaction
Outcomes	Incidence Rate Ratio (IRR) (95% CI)	*p value*	*p value*
Number of associated features of binge eating	1.005 (.861, 1.174)	.940	.761
Binge eating episodes	.942 (.756, 1.174)	.598	.621

### Hypothesis 2: The association between depressive symptoms and binge eating behaviours and cognitions both through the antenatal and postnatal period

In the univariable model, a one-unit increase in depressive symptom scores was associated with greater number of features associated with binge eating (Incidence Rate Ratio (IRR)= 1.092; 95% CI 1.082, 1.102). After adjustment for deprivation, baseline weight, lifetime features associated with binge eating and ethnicity there was still strong evidence for this associations albeit its magnitude was reduced (IRR = 1.030; 95% CI 1.019 to 1.041).

There was evidence of an association between greater depressive symptoms and higher number binge eating episodes (IRR = 1.100; 95% CI 1.085, 1.115) which was maintained after adjusting for deprivation, baseline weight, lifetime binge eating episodes and ethnicity albeit the magnitude was reduced (IRR = 1.031; 95% CI 1.015, 1.048). For every 1 unit increase in EPDS score there was an associated 1.100 increased risk for number of weekly binge eating episodes, decreasing to 1.031 when accounting for covariates (Table 4). 31.99% of variance in features associated with binge eating in the univariable model was accounted for by time point, increasing to 43.52% in the multivariable model. Evidence of positive associations between depressive symptoms and both greater number binge eating episodes and associated feature of binge eating supports hypothesis 2.

**Table 4:** Poisson multilevel models, nested within time point, for number of features associated with binge eating. b Adjusting for: Baseline weight, ethnicity and IMD, lifetime number of features associated with binge eating. Poisson multilevel models, nested within time point, for number of binge eating episodes. b Adjusting for: Baseline weight, ethnicity, IMD and lifetime number of binge eating episodes. Reporting IRR and 95%CI.

	**Number of features associated with binge eating**			
**Exposure**	**Univariable model**		**Multivariable model** ^**b**^	
	**IRR (95% CI)**	***p value***	**IRR (95% CI)**	***p value***
**EPDS score**	1.092 (1.082, 1.102)	<.0001	1.030 (1.019, 1.041)	<.0001
**Time point**	.948 (.527, 1.707)	.859	.905 (.478, 1.714)	.760
	**Number of binge eating episodes**			
**Exposure**	**Univariable model**		**Multivariable model** ^**b**^	
	**IRR (95% CI)**	***p value***	**IRR (95% CI)**	***p value***
**EPDS score**	1.100 (1.085, 1.115)	<.001	1.031 (1.015, 1.048)	<.0001
**Time point**	.963 (.500, 1.855)	.910	.916 (.463, 1.809)	.799

### Hypothesis 3- The effect of binge eating during pregnancy has on both gestational weight gain and birth weight

There was evidence of an association between higher number of features associated with binge eating and greater GWG (coefficient = .680; 95%CI .490, .869, p < .0001) which was robust to adjustment for deprivation, baseline weight, ethnicity, IMD score, depressive symptoms, lifetime features associated with binge eating and lifetime binge eating episodes score (Coefficient = .614; 95%CI .264, .964, p < .001). There was evidence of an association between number of binge eating episodes and GWG in unadjusted (coefficient = 1.264, 95%CI: .881, 1.646, p < .0001) but not in the adjusted model (coefficient = .172, 95%CI: −.531, .876, p = .631). There was no evidence of an association between greater number of binge eating episodes (unadjusted coefficient = 24.055, 95%CI: −29.553, 77.663, p = .379; adjusted coefficient = −48.258, 95%CI: −149.105 to 52.589, p = .348) or features associated with binge eating (unadjusted coefficient = 17.053, 95%CI: −9.583, 43.689, p = .210; adjusted coefficient = 5.266, 95%CI: −50.183, 60.714, p = 0.852) on birth weight (Table 5). Findings partially support hypothesis 3 that increases in binge eating behaviours and cognitions during pregnancy could lead to increased GWG.

**Table 5:** Linear regression multi-level models for Birth weight, Gestational weight gain at 28 and 36 weeks respectively, nested within timepoint and participant. a Adjusting for: Baseline weight, ethnicity, IMD, depression, life time binge endorsement and life time binge loss of control. b Adjusting for: Baseline weight, ethnicity, IMD, depression, life time binge endorsement and life time binge loss of control. Reported unstandardised coefficients and 95%CI.

	Birth weight (in grams)				Gestational weight gain in grams			
	Univariable model		Multivariable model^a^		Univariable model		Multivariable model^b^	
Exposure	Coefficient (95% CI)	*p value*	Coefficient (95% CI)	*p value*	Coefficient (95% CI)	*p value*	Coefficient (95% CI)	*p value*
**Number of features associated with binge eating**	17.053 (−9.583, 43.689)	*.210*	5.266 (−50.183, 60.714)	*.852*	.680 (.49ffffff0,.869)	*<.0001*	.614 (.264,.964)	*.001*
**Number of binge eating episodes**	24.055 (−29.553, 77.663)	*.379*	−48.258 (−149.105, 52.589)	*.348*	1.264 (.881, 1.646)	*<.0001*	.172 (−.531,.876)	*.631*

## 4. Discussion

In contrast with our hypothesis, we found no evidence of an effect of the UPBEAT lifestyle intervention in reducing binge eating behaviours during the antenatal period. In line with our hypotheses, however, we found that women who had greater depressive symptoms had higher level of binge eating episodes and features associated with binge eating across the perinatal period. This longitudinal analysis in a cohort of pregnant women living with obesity also provides evidence to support an association between features associated with binge eating behaviours and GWG but not birthweight.

The UPBEAT intervention appeared to have no effect on binge eating episodes and features associated with binge eating within the cohort. However, there is evidence to suggests that lifestyle interventions may be effective at reducing binge eating in women with BMI in overweight [54] and obese ranges [55,56]. These equivocal findings may be due to the UPBEAT intervention not being specifically designed to reduce binge eating in this population, similarly it has been found to have no effect on physical activity (Hayes et al., 2014), yet has been shown to improve diet (Flynn et al., 2016) and reduce GWG (Poston et al., 2015). Previous work has highlighted the need for effective lifestyle interventions for women living with obesity [57] with a strong focus on psychological treatments for BED [58]. For individuals living with obesity and BED they are recommended to have cognitive-behavioural therapy [59] or interpersonal psychotherapy [60], yet there are high rates of attrition for such treatment routes, for both individuals living with obesity and/or ED [61,62]. Given the complex nature of obesity and BED their prevention and treatment may be improved through not just targeting weight but also psychological aspects [63]. Psychoeducational interventions showing promising findings to reduce attrition and increase engagement in this particular population while treating BED [64].

Given the evidence for the association between depressive symptoms, features associated with binge eating behaviours and binge eating episodes in the perinatal period, physical activity centred interventions may be more suitable. Studies have found that small scale exercise-based interventions may improve depressive symptoms during pregnancy [65]. Further work is required given how UPBEAT used both physical activity and diet components and did not impact binge eating behaviours, similarly there is no effect on the main trial outcome of diabetic outcomes or depressive symptoms [36,48,66]. If the association between depressive symptoms and binge eating that we observe in this study is causal, reducing maternal depression could have positive effects in reducing binge eating behaviours. There is also good evidence supporting the effectiveness of psychological treatments, such as cognitive behavioural therapy (CBT) in the treatment of BED [67] and depression (Linde et al., 2015; Hollon, 2016), introduction of psychological therapies may improve both binge eating behaviours and depression within pregnant populations living with obesity. However, RCTs of such interventions are required to determine efficacy within pregnancy. No data is currently available to disentangle the effect of specific intervention components of UPBEAT on the participants’ mental health, which future research should address.

An association between depressive symptoms, features associated with binge eating behaviours and binge eating episodes during the perinatal period in pregnant women living with obesity is in line with previous research [1,12,68,69]. Evidence from pregnant populations suggests that individuals experiencing depressive symptoms are predisposed to disordered eating during pregnancy [1,68]. Pregnancy is a period of profound change including, endocrine and hormonal, body transformations, and psychological adjustments all of which have been linked to disordered eating and mood difficulties [70,71]. Thus, disordered eating may illustrate food-related coping mechanisms during the perinatal period [1,72,73].

Current findings demonstrated evidence of an association between binge eating behaviours and binge eating episodes on GWG. Following adjustment for depressive symptoms, features associated with binge eating behaviours, but not number of binge eating episodes, were drivers of excessive GWG. Binge eating episodes may not influence GWG due providing a too simplistic account of eating behaviour, not taking account dietary patterns or psychological factors that influence weight gain (Ferreira et al., 2022; Knoph Berg et al., 2011). Increased GWG can have significant implications for maternal and perinatal outcomes including increased birthweight, hypertension, caesarean section and non-routine maternal hospitalisation [74–76]. The effects of increased GWG can extend into the postnatal period, with excessive GWG being associated with higher risk of postpartum depression [77]. Qiu et al. (2022), found significant heterogeneity in outcomes, with recommendations to investigate the underlying psycho-biological mechanisms between GWG and postnatal depression, particularly in respect to postnatal weight retention [78].

### Strengths and limitations

The large sample represents a key strength of our study, enabling pregnancy outcomes such as GWG to be assessed with increased statistical power and precision. A unique strength of this analysis is that we were able to adjust for previous binge eating behaviours for all hypotheses, which is a limitation of previous literature (Keränen et al., 2009; LaRose et al., 2014; Nurkkala et al., 2015).

Our study also had a number of limitations. We could not assess whether individuals had a formal diagnosis for BED at all time points. Although general population samples show that the epidemiology of binge eating behaviours and that of BED is similar, it is possible that our results might not generalise to those with a BED diagnosis. Future research should therefore focus on investigating these research questions in those with BED.

Attrition was a limitation of this study, and was higher in the postnatal period, while maximum likelihood testing was applied for the antenatal period, attrition was too high to include the postnatal period. Future research should address this given how postnatal BED is influenced by antenatal eating behaviours [69,73,79]. Although we adjusted for key observed confounders, we cannot exclude residual confounding factors such as stressful events and lifestyle factors, which may have influenced both binge eating behaviours and depression. We were not able to investigate whether the effect of the UPBEAT intervention in hypothesis one differed according to baseline binge eating behaviours as our analyses would likely have been underpowered potentially resulting in type II error. While our analysis incorporated a measure of socio-economic status (SES; IMD) this was measured at an area-level, future work should endeavour to analyse SES using an individual measure to improve precision when adjusting for confounding [80].

Since participation into the trial was limited to women with higher BMI, we cannot exclude that collider (a type of selection) bias might explain why we did not find evidence of an association between binge eating and/or loss of control eating and selected birth outcomes (e.g., birthweight), which has been observed in other studies [23]. If women with higher BMI are more likely to experience binge eating [81] and to have children with higher birthweight [82,83], the effect of collider bias could be to drive the association under study towards the null. Replication of these findings in large general population studies including women across the BMI spectrum is therefore needed.

This analysis provides a new understanding of the association between mental health and ED behaviours. Future research could examine this relationship in populations with a clinical diagnosis of a mental health condition, where the relationship between binge eating and depression for example may be stronger.

## 5. Conclusions

We found no evidence that the UPBEAT intervention reduced binge eating behaviours during the antenatal period. This analysis provides a new understanding of the relationship between depression and binge eating behaviours during pregnancy among women living with obesity, which may be able to identify women who are clinically at risk of developing binge eating behaviours. Establishing holistic (e.g., diet, exercise and mental health) interventions to address mental health and eating behaviours in pregnancy may influence both intervention adherence and long-term health during and after the perinatal period [84,85].

## Supporting information

S1 Table Prevalence of binge endorsement, binge loss of control, n (%) for each timepoint and each trial arm. A is lifetime binge eating behaviours, b binge eating behaviours in the previous month.
(DOCX)
