# Supplementary material for: The effect of lifestyle intervention and depression symptoms on binge eating and relation of binge eating to gestational weight gain and child birth weight in the UPBEAT cohort of pregnant women living with obesity
Source: PLoS One. 2025 Sep 18;20(9):e0332569. doi: 10.1371/journal.pone.0332569 (PMC12445464; doi:10.1371/journal.pone.0332569)
Supplement: S1 Table — A is lifetime binge eating behaviours, b binge eating behaviours in the previous month. (DOCX) [file pone.0332569.s001.docx]

**Supplementary table 1: Prevalence of binge endorsement, binge loss of control, n (%) for each timepoint and each trial arm. A is lifetime binge eating behaviours, b binge eating behaviours in the previous month.**

|  | | **Time Point** | | | | | | | | | |
| --- | --- | --- | --- | --- | --- | --- | --- | --- | --- | --- | --- |
|  |  | **Baseline^a^** | | | **28 Weeks^b^** | | **32 Weeks^b^** | | | **6 Weeks Post-partum^b^** | |
| **Binge eating variable** |  | **n(%)** | | | **n(%)** | | **n(%)** | | | **n(%)** | |
|  |  | **Control** | **Treatment** | | **Control** | **Treatment** | **Control** | **Treatment** | | **Control** | **Treatment** |
| **Number of binge eating episodes with loss of control present** | 0  1  2  3  4  **Yes**  **No**  N | 513 (37.89%)  71 (5.24%)  51 (3.77%)  31 (2.29%)  13 (.96%)  **166 (12.26%)**  **513 (37.89%)**  1354 | 511 (37.74%)  66 (4.87%)  58 (4.28%)  27 (1.99%)  13 (.96%)  **164 (12.10%)**  **511 (37.74%)** | | 521 (45.46%)  19 (1.66%)  16 (1.40%)  0 (0%)  2 (.17%)  **37 (3.23%)**  **521 (45.46%)**  1146 | 522 (48.17%)  20 (1.75%)  9 (.79%)  6 (.52%)  1 (.09%)  **36 (3.15%)**  **522 (48.17%)** | 431 (44.52%)  20 (2.07%)  5 (.52%)  1 (.10%)  1 (.10%)  **27 (2.79%)**  **431 (44.52%)**  968 | 488 (50.41%)  15 (1.55%)  3 (.31%)  3 (.31%)  1 (.10%)  **22 (2.27%)**  **488 (50.41%)** | | 285 (40.31%)  30 (4.24%)  21 (2.97%)  10 (1.41%)  3 (.42%)  **64 (9.04%)**  **285 (40.31%)**  707 | 279 (49.46%)  35 (4.95%)  23 (3.25%)  15 (2.12%)  6 (.85%)  **79 (11.17%)**  **279 (49.46%)** |
| **Number of binge eating behaviours endorsed by participant** | 0  1  2  3  4  5 | 484 (35.60%)  23 (1.69%)  38 (2.80%)  36 (2.65%)  49 (3.61%)  51 (3.76%) | 478 (35.20%)  28 (2.06%)  26 (1.91%)  49 (3.61%)  37 (2.72%)  59 (4.34%) | | 518 (45.12%)  3 (.26%)  12 (1.05%)  13 (1.13%)  7 (.61%)  6 (.52%) | 546 (47.56%)  7 (.61%)  9 (.78%)  12 (1.05%)  5 (.44%)  10 (.87%) | 430 (44.42%)  5 (.52%)  7 (.72%)  5 (.52%)  6 (.62%)  5 (.52%) | 482 (49.79%)  6 (.62%)  4 (.41%)  4 (.41%)  9 (.93%)  5 (.52%) | | 281 (39.69%)  10 (1.41%)  11 (1.55%)  12 (1.69%)  14 (1.98%)  21 (2.97%) | 271 (38.28%)  12 (1.69%)  19 (2.68%)  19 (2.68%)  18 (2.54%)  20 (2.82%) |
| **Endorsement of any binge eating behaviour**  **(score of 3 or more=yes)** | **Yes**  **No**  N | **136 (10.01%)**  **545 (40.13%)** | | **145 (10.68%)**  **532 (39.18%)** | **26 (2.26%)**  **533 (46.43%)** | **27 (2.35%)**  **562 (48.95%)** | **16 (1.65%)**  **442 (45.66%)** | | **18 (1.86%)**  **50.83%)** | **47 (6.63%)**  **302 (42.66%)** | **57 (8.05%)**  **302 (42.66%)** |
|  |  | 1358 | | | 1148 | | 968 | | | 708 | |

Supplementary Table1 Prevalence of binge endorsement, binge loss of control, n (%) for each timepoint and each trial arm. A is lifetime binge eating behaviours, b binge eating behaviours in the previous month.
